# Clinical utility of companion diagnostic biomarker results below the limit of detection in comprehensive genomic profiling of patients with advanced non-small cell lung cancer

**DOI:** 10.1093/oncolo/oyaf159

**Published:** 2025-06-23

**Authors:** Gerald Li, Stephanie B Greene, Baljinder Kaur, Rachel B Keller-Evans, Ryon P Graf, Brennan Decker, David L Smith, Richard S P Huang

**Affiliations:** Medical Affairs, Foundation Medicine, Inc., Boston, MA 02210, USA; Diagnostic Development, Foundation Medicine, Inc., Boston, MA 02210, USA; Diagnostic Development, Foundation Medicine, Inc., Boston, MA 02210, USA; Clinical Development, Foundation Medicine, Inc., Boston, MA 02210, USA; Medical Affairs, Foundation Medicine, Inc., Boston, MA 02210, USA; Diagnostic Development, Foundation Medicine, Inc., Boston, MA 02210, USA; Diagnostic Development, Foundation Medicine, Inc., Boston, MA 02210, USA; Clinical Development, Foundation Medicine, Inc., Boston, MA 02210, USA

**Keywords:** limit of detection, limit of blank, clinical utility, comprehensive genomic profiling

## Abstract

**Background:**

When the limit of blank (LoB) of comprehensive genomic profiling (CGP) for a given biomarker is acceptably demonstrated (ie, α ≤ 0.05 or LoB equal to zero), biomarkers detected below the assay limit of detection (LoD) can be reported with a high degree of confidence. However, it is unknown whether variants detected below LoD have clinical utility.

**Patients and Methods:**

This study used a de-identified nationwide (US-based) non-small cell lung cancer clinico-genomic database (CGDB) containing linked FDA-approved CGP testing from Foundation Medicine, Inc (FMI). We selected patients who received an FMI CGP report with an actionable biomarker detected below LoD. We assessed clinical utility among those patients who received an appropriately matched targeted therapy, defined as a real-world overall response rate exceeding a prespecified threshold of 30% based on historical chemotherapy response rates.

**Results:**

Among 129 patients who had a biomarker detected and reported below LoD, received the appropriate matched targeted therapy, and were assessed for response, partial or complete response was observed in 36/54 (67%, one-tailed 95% CI: >55%, *P* < .001) patients tested with a tissue-based CGP test and 54/75 (72%, one-tailed 95% CI: >62%, *P* < .001) patients tested with a liquid-based CGP test, both of which exceeded the prespecified threshold for clinical utility.

**Conclusions:**

Most patients who receive a targeted therapy matched to a companion diagnostic biomarker detected and reported below LoD demonstrate clinical benefit from that therapy. This clinical observation suggests actionable variants should continue to be reported when detected with FMI CGP tests.

**Implications for Practice**

Rigorous validation of Foundation Medicine’s comprehensive genomic profiling tests has demonstrated that biomarkers are not reported when absent from a specimen, ensuring confidence in any reported actionable biomarker. However, the clinical utility of biomarkers detected below the limit of detection (LoD) from formal validation studies was previously unproven. This study demonstrates the clinical utility of below LoD companion diagnostic biomarkers, as superior response rates were observed when such patients received matched targeted therapy compared to historical response rates for similar patients receiving chemotherapy. Foundation Medicine reports all companion diagnostic biomarkers that have been detected with confidence, including those below LoD, to provide clinicians with the information needed to prescribe the appropriate targeted therapies to those patients who will benefit most.

## Introduction

Analytical validation of next-generation sequencing (NGS) assays is essential to determine the safety and performance characteristics of a medical device, which is critical for clinical use. These studies routinely encompass numerous aspects of assay performance, such as limit of blank (LoB), limit of detection (LoD), precision, accuracy, and concordance with orthogonal assay results.

Clinically, LoB is typically determined with the highest apparent analyte concentration expected to be measured using replicates of a blank sample containing no analyte. To determine LoB for a comprehensive genomic profiling (CGP) test, multiple specimens known to be negative for the variant of interest are assayed individually at or near maximal assay input. Per industry standard, the false positive rate may not exceed 5% of the total specimen replicates assayed in order to claim a LoB of zero. And per industry standard a false positive rate ≤5% is acceptable to enable determination of LoD.

LoD is the minimum level of variant allele frequency (VAF) or supporting chimeric reads for rearrangements that can be detected with a defined degree of confidence. VAF is the proportion of sequencing reads that contain a specific variant relative to all sequencing reads for the given genetic loci for that sample. To determine LoD for a variant, a sample known to harbor the variant is serially diluted and assayed with multiple replicates per dilution level. The VAF (or number of supporting chimeric reads for rearrangements) of the variant in the lowest dilution level with a detection rate ≥95% (per industry standard) is defined as the variant LoD. Foundation Medicine, Inc. (FMI) used these approaches to determine the LoB and LoD of individual assays, details of which are published both in validation studies and on the technical labels of the respective assays.^1-4^

While LoD is a single value, detection or VAF is a continuous variable. Sensitivity decreases below LoD (ie, the confidence in variant detection below LoD decreases when the variant is present). However, it is possible to detect variants when present below the established LoD. When the LoB is reliably distinguished from the LoD, true positive variants at VAF below the LoD can be detected and reported in clinical specimens, all while avoiding false positives. Having established both LoB and LoD, including an LoB of zero,^3,4^ and taking into consideration other stringent quality control metrics (eg, VAF or supporting chimeric reads, depth of sequencing, read quality, tiering status, etc.), Foundation Medicine, Inc. (FMI) can detect variants below LoD with high confidence and report them.

While more recent FMI test reports include the VAF for detected variants, the variant LoD is not included directly in the report and is not utilized as a quality metric to determine whether a variant is reported. Instead, sample and sequencing quality metrics (eg, error rate, uniformity of coverage, median exon coverage, tumor content, copy number noise, contamination, and reference coverage) are utilized to set reporting thresholds that determine which genomic alterations appear on the physician-facing report. Clinical utility^5^ of treatment decisions associated with samples harboring below LoD variants, ie, whether the patient outcome is improved when a patient receives therapy matched to a below LoD biomarker on an FMI report, remains to be demonstrated. We sought to address this issue using a real-world cohort of patients with advanced NSCLC with linked clinical and genomic data. These data can inform the practice of reporting below LoD results and enable maximizing the number of patients who would benefit from appropriate matched targeted therapy.

## Materials and methods

### Study design and patient selection

The cohort consisted of patients diagnosed with advanced (Stage IIIB-IV or recurrent) non-small cell lung cancer (NSCLC) between January 2011 and December 2023 in the US-wide Flatiron Health-Foundation Medicine (FMI) de-identified clinico-genomic database (CGDB). De-identified data originated from approximately 280 US cancer clinics (~800 sites of care). Retrospective longitudinal clinical data were derived from electronic health records, comprising patient-level structured and unstructured data, curated via technology-enabled abstraction, and were linked to genomic data derived from FMI CGP tests by de-identified, deterministic matching.^6^

All patients in the cohort received an FMI CGP report reflecting positivity for a companion diagnostic (CDx) biomarker below the published LoD from the technical label for the respective assay (Table 1).^1,2^ For clarity, in all cases, the biomarker was detected below LoD, met stringent quality metrics, and was included in the clinician-facing report. More recent versions of the report also included the VAF, but in no cases was the LoD directly placed on the report. Due to the limited number of below LoD biomarker-positive patients in the CGDB, we included some patients tested with a comparable recent prior version of the FMI tissue assay. Additionally, patients must have received the matching targeted therapy as the monotherapy or combination specified on the diagnostic label.

**Table 1.** Companion diagnostic biomarkers studied, with published limits of detection for FoundationOne®CDx and FoundationOne®Liquid CDx and their associated targeted therapies.

CDx biomarker	FoundationOne®CDx LoD	FoundationOne®CDx therapies	FoundationOne®liquid CDx LoD	FoundationOne®liquid CDx therapies	Notes
*EGFR* Exon 19 deletion	VAF: 5.1%	Afatinib, Dacomitinib, Erlotinib, Gefitinib, Osimertinib	VAF: 0.45%	Erlotinib, Gefitinib, Osimertinib	
*EGFR* Exon 21 L858R	VAF: 2.4%	Afatinib, Dacomitinib, Erlotinib, Gefitinib, Osimertinib	VAF: 0.64%	Erlotinib, Gefitinib, Osimertinib	
*EGFR* Exon 20 T790M	VAF: 2.5%	Osimertinib	N/A	N/A	No liquid CDx
*BRAF* V600E	VAF: 2.0%	Binimetinib + Encorafenib, Dabrafenib + Trametinib	VAF: 0.86%	Binimetinib + Encorafenib	
*MET* Exon 14 SNVs	VAF: 2.9%	Capmatinib	VAF: 0.40%	Capmatinib	*MET* exon 14 skipping was counted separately but reported in aggregate.
*MET* Exon 14 insertion and deletion	VAF: 5.7%	Capmatinib	VAF: 0.28%	Capmatinib	*MET* exon 14 skipping was counted separately but reported in aggregate.
*ALK* rearrangements	Chimeric reads: 15.54	Alectinib, Ceritinib, Crizotinib	VAF: 0.68%	Alectinib	
*ROS1* fusions	Chimeric reads: 11.85	Entrectinib	VAF: 1.30%	Entrectinib	
*NTRK1* rearrangements	Chimeric reads: 24.55	Entrectinib, Larotrectinib	VAF: 0.75%	Entrectinib	*NTRK* is counted separately and reported in aggregate.
*NTRK2* rearrangements	Chimeric reads: 24.16	Entrectinib, Larotrectinib	N/A	Entrectinib	*NTRK* is counted separately and reported in aggregate.
*NTRK3* rearrangements	Chimeric reads: 14.65	Entrectinib, Larotrectinib	VAF: 0.68%	Entrectinib	*NTRK* is counted separately and reported in aggregate.
*RET* fusions	Chimeric reads: 8.75	Selpercatinib	N/A	N/A	No liquid CDx

IRB approval of the study protocol was obtained prior to the study conduct and included a waiver of informed consent.

### Comprehensive genomic profiling

Hybrid capture-based NGS assays were performed on patient tumor or blood specimens in a Clinical Laboratory Improvement Amendments–certified, College of American Pathologists-accredited laboratory (FMI, Cambridge, MA). FoundationOne®CDx^1,3^ and FoundationOne®Liquid CDx^2,4^ were performed as previously described. The tissue CGP cohort included specimens tested with FoundationOne®, which has very high concordance and similar operating characteristics to FoundationOne®CDx, as demonstrated during analytical validation of the latter.^1^ All these assays have previously been shown to have LoB equivalent to zero.^1-4^

### Endpoints

Real-world response to therapy (rwR) was manually abstracted from unstructured clinician documentation as previously described.^7^ In brief, abstractors were instructed to review all relevant documents, including clinician notes and radiology reports. For each instance of radiologic response assessment, abstractors then captured the treating clinician’s assessment or interpretation of the radiographic scans and mapped them to the following categories, according to the change in disease burden observed: complete response, partial response, stable disease, progressive disease, and not documented or indeterminate response.^7,8^ Unlike Response Evaluation Criteria in Solid Tumors, rwR anchors to clinician interpretation of radiographic assessments, which better reflects response assessment in routine clinical practice outside of clinical trials, rather than an objective comparison of measurements of target lesions. rwR has been validated by comparison to data from the IMpower132 clinical trial.^8^ The real-world overall response rate (rwORR) was calculated as the percentage of patients in whom complete response or partial response was observed during the earliest oncologist-defined, rule-based line of therapy matching the below LoD biomarker among those who were evaluable for rwR.

The critical rwORR for targeted therapy to demonstrate clinical benefit was prespecified at 30% based on a literature review showing targeted therapies typically have response rates >60% while chemotherapy typically has response rates ~30%,^9-16^ combined with a preliminary power calculation performed without accessing the rwR variable.

### Statistical analysis

All analyses were conducted using R version 4.4.0.

In the primary analysis, rwORR was compared against the prespecified threshold using a one-tailed 95% Clopper-Pearson binomial confidence interval, with the alternate hypothesis of the observed rwORR exceeding the prespecified threshold. This aligns with our hypothesis that patients with a tumor that is positive for a below LoD biomarker will experience clinical benefit from receiving the appropriate matched targeted therapy.

A statistical analysis plan was prospectively declared, detailing cohort selection criteria, analytical methodology, and pre-specification of the critical rwORR based on prior literature, as described in *Endpoints*. The plan called for the response rate to be reported in aggregate across all biomarkers, with additional sensitivity analyses to confirm results were consistent across individual biomarkers. A prespecified coprimary analysis repeated the primary and sensitivity analyses but considered only patients who received matched targeted therapy in their first advanced line of therapy since first-line response rates are typically better than for the same therapy in later lines.^11,12^ The plan also evaluated the feasibility of time-to-event endpoints like time to next treatment but these were deemed to be unfeasible due to small cohort size.

## Results

**Figure 1.**
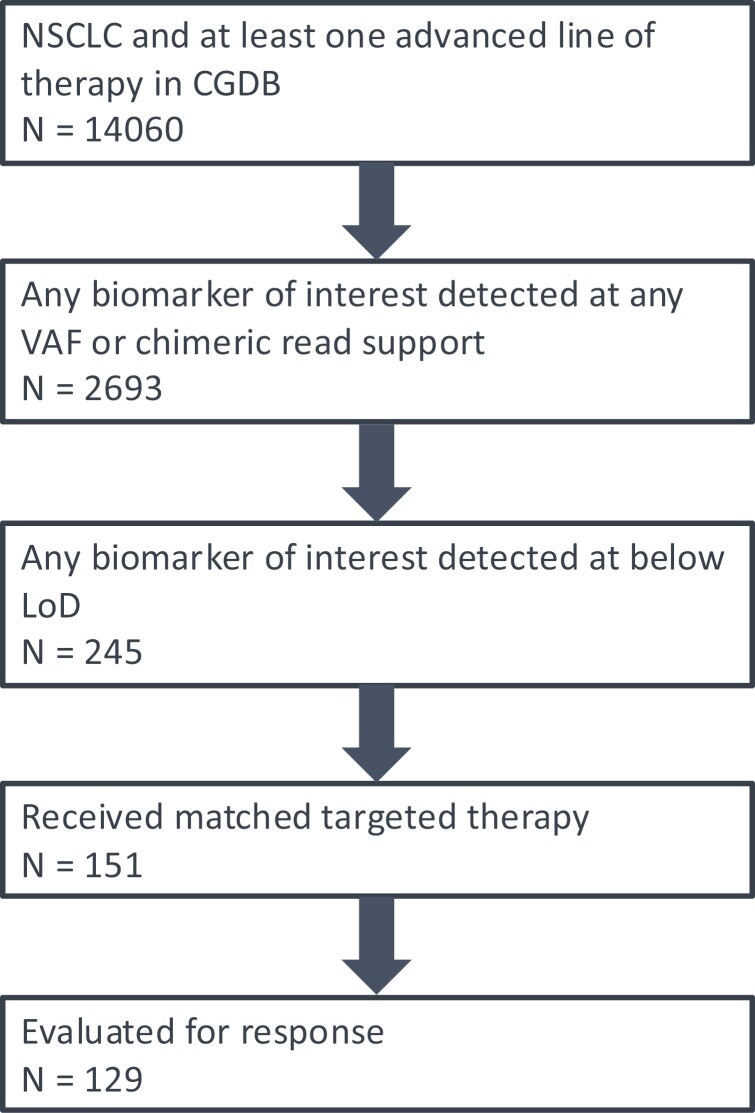
CONSORT diagram showing the number of patients satisfying each of the study’s inclusion criteria.

Of 14 060 patients with advanced NSCLC in the CGDB and who received at least one line of therapy, 2693 (19%) patients received a CGP report detecting at least one CDx biomarker from Table 1 and of these, 245 (9.1% of positive results) were below LoD. Of these, 62% (151: 67 in tissue and 85 in liquid, including one patient with both) received matched targeted therapy in the advanced setting. Of these, 129 (85%) were evaluated for response while receiving said therapy (Figure 1).

**Figure 2.**
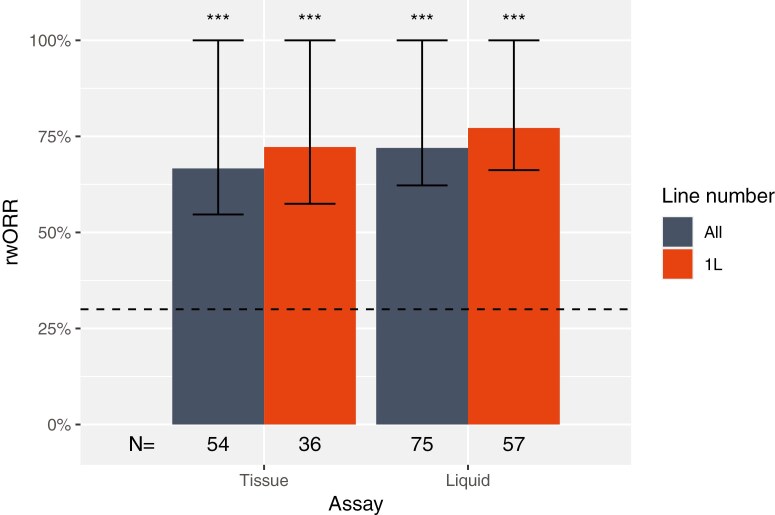
Real-world overall response (rwORR) rates for patients with targetable biomarkers detected below the validated LoD of a tissue or liquid CGP assay who received the corresponding matched targeted therapy in either the first or any line of therapy. The prespecified threshold of 30% is shown as a dotted line for reference and the one-tailed 95% CI is represented. The detailed breakdown by biomarker is presented in Supplementary Figures 1 and 2. Stars denote *P*-value ranges for the comparison of the one-tailed 95% confidence intervals with the prespecified threshold of 30%: *P* < .1, *: *P* < .05, **: *P* < .01, ***: *P* < .001.

Among patients with a biomarker detected below LoD in tissue CGP and who received matched targeted therapy, the rwORR was 67% (responses noted in 36 out of 54 patients assessed, one-tailed 95% CI: >55%, *P* < .001; Figure 2), exceeding the prespecified threshold of 30%. All biomarkers with at least 10 patients had a one-tailed 95% CI lower bound on the rwORR that exceeded the prespecified threshold of 30% (Supplementary Figure 1). The aggregate rwORR was more favorable (72%, responses noted in 26 out of 36 patients assessed, 95% CI: >57%, *P* < .001) when restricting the analysis to patients who received matched targeted therapy in the first line.

Among patients with a below LoD biomarker detected in liquid CGP and who received matched targeted therapy, the rwORR was 72% (responses noted in 54 out of 75 patients assessed, one-tailed 95% CI: >62%, *P* < .001; Figure 2). rwORR both in aggregate and for all biomarkers with at least 10 patients had one-tailed 95% CI lower bounds that exceeded the prespecified threshold of 30% (Supplementary Figure 2). The aggregate rwORR was more favorable (77%, responses noted in 44 out of 57 patients assessed, 95% CI: >66%, *P* < .001) when restricting the analysis to patients who received matched targeted therapy in the first line.

For reference, we repeated our analyses on all patients with the biomarkers of interest and who received matched targeted therapy, without filtering on LoD, and found an aggregate rwORR > 70% and lower bounds on the confidence intervals >68% (Supplementary Figure 3).

## Discussion

To the best of our knowledge, this is the first systematic evaluation of clinical responses in patients where actionable biomarkers were detected and reported below the validated LoD associated with 95% sensitivity in a CGP assay with an acceptably demonstrated LoB. Our analysis reveals: 1. In standard practice settings, CGP reports with below LoD biomarkers are uncommon (9.1% of reports with at least one biomarker detected or 1.7% of all reports) but such patients routinely receive matched targeted therapies (Figures 1 and 2 ). When these results are delivered and acted upon, patient outcomes are improved by a significant margin over expectations from literature reports of similar patients on chemotherapy.

Across all studied biomarkers, patients with below LoD biomarker reports who received matched targeted therapy observed a rwORR of roughly 70% in aggregate and confidence interval lower bounds >50%, far exceeding the prespecified threshold of 30% that was chosen on the basis of a broad literature review and consistent with response rates observed both in clinical trials for these drugs^9-16^ and in the broader group of patients in our dataset who had any of these biomarkers detected at any level, including above LoD. This result was also consistent across all individual biomarker cohorts with nominal levels of statistical power (≥10 patients). These results support the continued reporting of below LoD biomarker detections to maximize the number of patients who may benefit from matched targeted therapy.

### Limitations

Observational and/or retrospective analyses are more prone to false discovery than prospective randomized trials, due to multiple hypothesis testing and potential imbalances between groups. We used a prospectively declared statistical analysis plan to prespecify hypotheses and analyses to reduce the risk of false discovery and made use of an outcome measure (rwORR) that was closely tied to the effect of the drug and less prone to effect confounding than time-to-event measures like PFS or OS.

Many factors can contribute to a low-VAF variant detection, including low tumor purity of a tissue specimen, low shed tumor for a liquid biopsy, and subclonality, among others. Some causes, like subclonality, may be expected to reduce sensitivity to targeted therapy, while others like low tumor purity might not. This study did not attempt to distinguish the various root causes, so it captures an aggregate or “average” effect. Additionally, as some of these factors contributing to low VAF may be assay-specific, the results here are specific to recent FMI CGP assays and not generalizable to all NGS tests. There may be disease-specific characteristics that impact the clinical utility of low-VAF biomarkers in NSCLC. While this study demonstrated that robust quality metrics and LoB equivalent to 0 ensure high specificity of variants detected below LoD, it is unclear whether below LoD biomarker detections in other tumor types, would have the same clinical utility due to potential biological differences between tumor types.

## Conclusion

These results suggest that about 9% of actionable biomarkers on FMI CGP reports are detected at below LoD. While uncommon, below LoD CDx biomarker detections by recent FMI CGP assays predict clinical benefit from matched targeted therapy. Therefore, FMI’s current practice of including on the report all biomarkers detected with sufficient confidence, including those below LoD, to maximize the number of patients who may benefit from matched targeted therapy, subject to stringent quality control to minimize false positives, is supported by this analysis and should continue. Acknowledging that there are additional NGS assays marketed for use to inform the treatment of patients with cancer, the oncology community would be well-served by evaluating the clinical utility of below LoD biomarker results from other assays.

## Supplementary Material

oyaf159_suppl_Supplementary_Figures

## Data Availability

The data that support the findings of this study have been originated by Flatiron Health, Inc., and Foundation Medicine, Inc. Requests for data sharing by license or by permission for the specific purpose of replicating results in this manuscript can be submitted to dataaccess@flatiron.com and cgdb-fmi@flatiron.com.

## Notes

This research did not receive any specific grant from funding agencies in the public, commercial, or not-for-profit sectors.
