# Supplementary material for: Clinical utility of companion diagnostic biomarker results below the limit of detection in comprehensive genomic profiling of patients with advanced non-small cell lung cancer
Source: Oncologist. 2025 Jun 23;30(6):oyaf159. doi: 10.1093/oncolo/oyaf159 (PMC12205977; doi:10.1093/oncolo/oyaf159)
Supplement: oyaf159_suppl_Supplementary_Figures [file oyaf159_suppl_supplementary_figures.pdf]

Supplementary figures for:

Clinical utility of companion diagnostic biomarker results below the limit of detection in comprehensive genomic profiling of patients with advanced non-small cell lung cancer

Gerald Li, Stephanie B. Greene, Baljinder Kaur, Rachel B. Keller-Evans, Ryon P. Graf, Brennan Decker, David L. Smith, Richard S.P. Huang

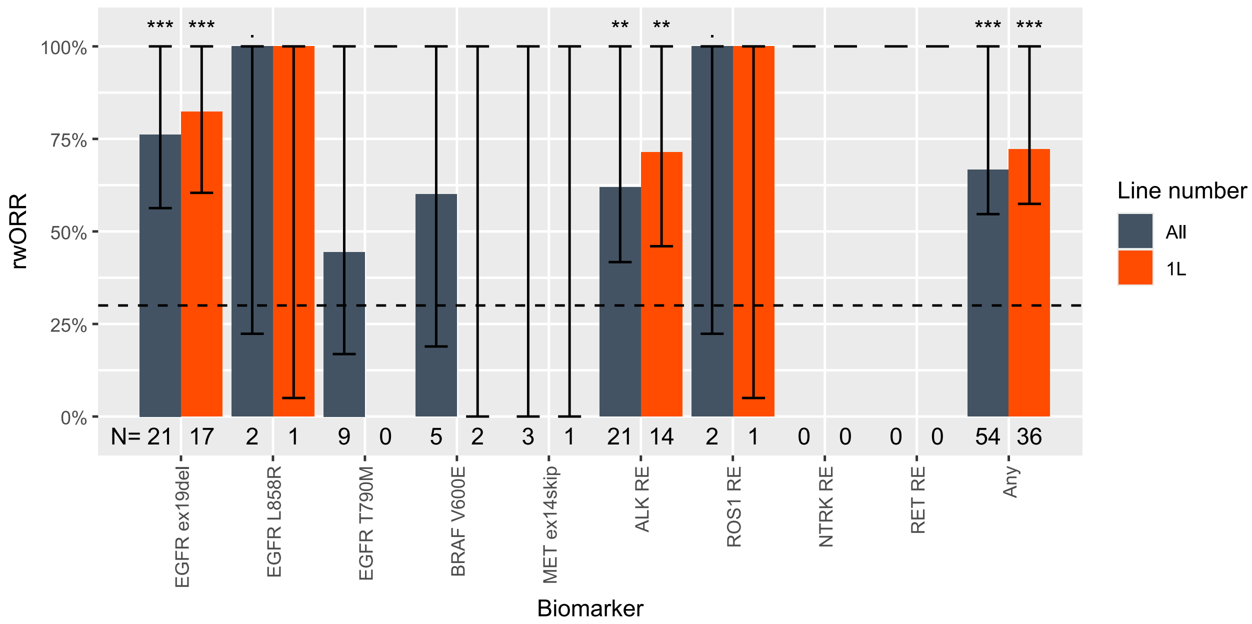

Supplementary Figure 1: Real-world overall response rates (rwORR) with one-tailed 95% confidence intervals for patients who received a tissue CGP test reporting each of the biomarkers of interest below LoD and received the corresponding matched targeted therapy in either the first or any advanced line of therapy. The prespecified threshold of 30% is shown as a dotted line for reference. Stars denote *P*-value ranges for the comparison of the one-tailed 95% confidence intervals with the pre-specified threshold of 30%: . : $P < .1$, * : $P < .05$, ** : $P < .01$, *** $P < .001$.

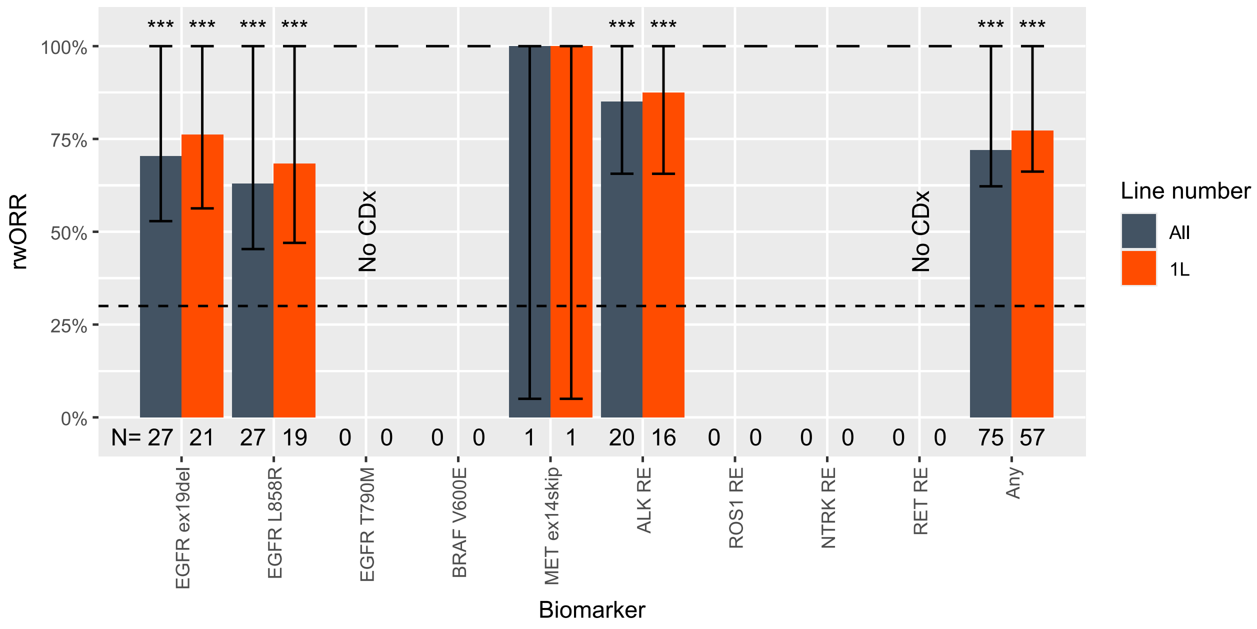

Supplementary Figure 2: Real-world overall response rates (rwORR) with one-tailed 95% confidence intervals for patients who received a liquid CGP test reporting each of the biomarkers of interest below LoD and received the corresponding matched targeted therapy in either the first or any advanced line of therapy. The prespecified threshold of 30% is shown as a dotted line for reference. Stars denote *P*-value ranges for the comparison of the one-tailed 95% confidence intervals with the pre-specified threshold of 30%: . : *P* < .1, * : *P* < .05, ** : *P* < .01, *** *P* < .001.

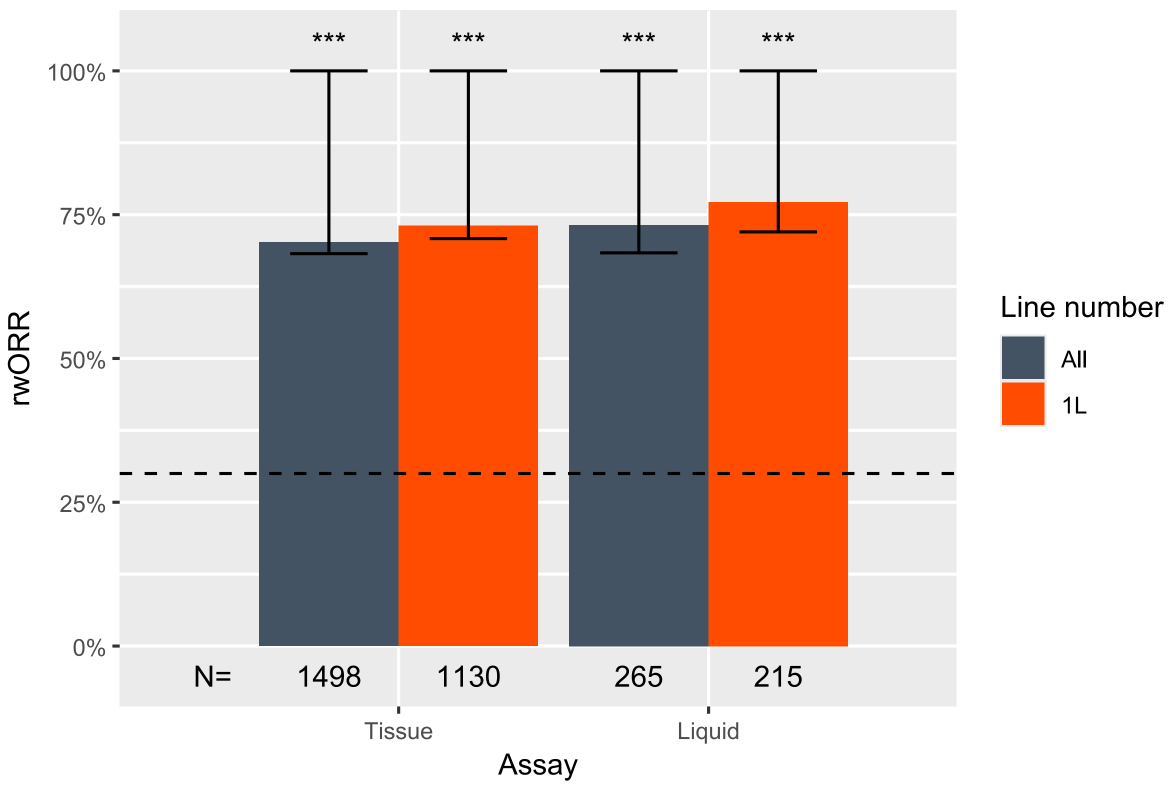

Supplementary Figure 3: Real-world overall response rates (rwORR) with one-tailed 95% confidence intervals for patients who received a tissue or liquid CGP test reporting any of the biomarkers of interest at any VAF/chimeric read support and received the corresponding matched targeted therapy in either the first or any line of therapy. The pre-specified threshold of 30% is shown as a dotted line for reference. Stars denote *P*-value ranges for the comparison of the one-tailed 95% confidence intervals with the pre-specified threshold of 30%: . : *P* < .1, * : *P* < .05, ** : *P* < .01, *** *P* < .001.
